# Brain Oscillations Elicited by the Cold Pressor Test: A Putative Index of Untreated Essential Hypertension

**DOI:** 10.1155/2017/7247514

**Published:** 2017-05-09

**Authors:** Christos Papageorgiou, Efstathios Manios, Eleftheria Tsaltas, Eleni Koroboki, Maria Alevizaki, Elias Angelopoulos, Meletios-Athanasios Dimopoulos, Charalabos Papageorgiou, Nikolaos Zakopoulos

**Affiliations:** ^1^Department of Clinical Therapeutics, National and Kapodistrian University of Athens, Medical School, Athens, Greece; ^2^1st Department of Psychiatry, National and Kapodistrian University of Athens, Medical School, “Eginition” Hospital, 115 28 Athens, Greece; ^3^University Mental Health Research Institute (UMHRI), Athens, Greece

## Abstract

**Objective:**

Essential hypertension is associated with reduced pain sensitivity of unclear aetiology. This study explores this issue using the Cold Pressor Test (CPT), a reliable pain/stress model, comparing CPT-related EEG activity in first episode hypertensives and controls.

**Method:**

22 untreated hypertensives and 18 matched normotensives underwent 24-hour ambulatory blood pressure monitoring (ABPM). EEG recordings were taken before, during, and after CPT exposure.

**Results:**

Significant group differences in CPT-induced EEG oscillations were covaried with the most robust cardiovascular differentiators by means of a Canonical Analysis. Positive correlations were noted between ABPM variables and Delta (1–4 Hz) oscillations during the tolerance phase; in high-alpha (10–12 Hz) oscillations during the stress unit and posttest phase; and in low-alpha (8–10 Hz) oscillations during CPT phases overall. Negative correlations were found between ABPM variables and Beta2 oscillations (16.5–20 Hz) during the posttest phase and Gamma (28.5–45 Hz) oscillations during the CPT phases overall. These relationships were localised at several sites across the cerebral hemispheres with predominance in the right hemisphere and left frontal lobe.

**Conclusions:**

These findings provide a starting point for increasing our understanding of the complex relationships between cerebral activation and cardiovascular functioning involved in regulating blood pressure changes.

## 1. Introduction

Hypertension is a leading risk factor for cardiovascular disease and a major contributor to healthcare costs worldwide [1]. Given that autonomic nervous system (ANS) activity modulates transient changes in cardiovascular function, autonomic dysfunction has been implicated in the pathogenesis of essential hypertension (EH) [2]. Increased sympathetic activity [3] combined with parasympathetic inhibition may contribute to increased cardiac activity and/or peripheral vascular resistance and thereby to the early development of hypertension [4], although patients with borderline to mild hypertension often show normal vascular resistance at rest.

The central nervous system (CNS) has also been implicated in the aetiology and maintenance of some forms of EH. The CNS is a target of the disease which, if untreated, progresses to blood pressure (BP) levels threatening the integrity of cerebral vessels, potentially inducing stroke [5].

Considerable evidence supports the connection between pain perception and BP regulation. It has been proposed that acute BP increases may reduce pain, thus establishing hypertension through instrumental learning [6]. Hypoalgesia has been noted in animals and humans with high BP [7], but the issue of whether it precedes or follows hypertension remains equivocal.

The layman concept that stress can cause hypertension still lacks strong empirical support. A review of studies examining the relationship of stress and hypertension [8] concluded that environmental measures of stress, such as natural disasters, unsafe neighborhood conditions, and work stress, were related to increased BP. In contrast, minimal association was noted between self-rated stress and BP.

Pain sensations, consisting of sensory, affective, and cognitive experiences, modulate EEG oscillations across a wide range of frequency bands, presumably reflecting the mechanisms involved in cortical activation and inhibition [9, 10]. Several studies have investigated the impact of experimental pain on human EEG. Most of these were based on the cold pressure test (CPT). There is consensus that the CPT produces decreased alpha EEG activity. Similarly, Beta and Theta bands activity has previously been reported to increase during CPT [11, 12].

Taking into consideration the issues presented above, the current study attempted an integrative approach to the factors interacting in the setting of hypertension. The objective was to compare well-characterised, untreated hypertensives and matched normotensive controls in terms of (i) arterial blood pressure variables (24-hour ambulatory blood pressure monitoring (ABPM)), (ii) CNS electrophysiological responsiveness (EEG), and (iii) behavioural responsiveness (pain perception and tolerance) under exposure to sympathoexcitatory stress and pain induced by the CPT.

Although the impact of experimental pain on EEG has attracted experimental interest, the existing available data do not warrant the formulation of specific hypotheses regarding the relationship between brain oscillations and newly diagnosed, untreated hypertension. Therefore this axis of our design has an exploratory character.

## 2. Methods

### 2.1. Study Population

The hypertensive (HT) group consisted of 22 newly diagnosed untreated hypertensives (11 men, 11 women; mean age = 50.59 ± 11.45 years) referred to the hypertensive centre of the Department of Clinical Therapeutics (Athens University, Greece). The normotensive control (NT) group included 18 healthy volunteers (8 men and 10 women, mean age = 51.72 ± 8.33 years). All 40 participants were ambulatory and the two groups were matched for sex, age, and body mass index. They all fulfilled the following inclusion criteria: (1) no previous antihypertensive treatment; (2) absence of clinical signs or laboratory evidence of hypertension-related complications (coronary artery disease, heart failure, cerebrovascular disease, renal insufficiency, or peripheral artery disease) or of secondary causes of arterial hypertension; (3) absence of any other systematic disease; (4) absence of psychiatric disorders or psychiatric medication; (5) at least three valid BP measurements per hour over 24 hr ambulatory blood pressure monitoring (ABPM: 75% successful measurements). The cohort was initially evaluated by 24-hour ABPM and subjects were divided into 2 groups in terms of their ABPM measurements objectively on the basis of this measurement. Individuals with 24-hour ambulatory BP <130/80 mmHg were placed in the NT group; individuals with 24-hour ambulatory BP ≥130/80 mmHg were placed in the HT group. All subjects gave their informed consent for participation in the study, and the study protocol was approved by the hospital ethics review committee to ensure that the procedures followed were in accordance with the institutional guidelines.

### 2.2. Procedure

Participants were instructed to abstain from alcohol, cigarette smoking, coffee/tea, and exercise for at least 30 minutes prior to testing. The study flow diagram can be seen in Table 1.

#### 2.2.1. The 24-Hour ABPM Measurement [13, 14]

24-hour ABPM was conducted on all subjects on a usual working day by means of the Spacelabs 90217 ambulatory blood pressure monitor (Spacelabs Inc., Redmond, Wash). The appropriate sized cuff was placed around the nondominant arm and 3 consecutive blood pressure determinations were recorded along with sphygmomanometric measurements to verify that there was no difference greater than 5 mmHg on the average of the 2 sets of values. Throughout the 24-hour monitoring readings were obtained automatically at 15-minute intervals and all subjects had at least 3 valid readings per hour. The resulting 80 to 96 pairs of systolic and diastolic BP readings per recording with the corresponding recording time were used to calculate blood pressure derivatives. All subjects were instructed to maintain their usual daytime activities between 6:00 AM and 10:00 PM and rest-sleep between 10:00 PM and 6:00 AM [13]. In this context it is useful to outline the time rate (TR) of BP variation. The TR of BP variation was defined as the first derivative of the BP values against time and was calculated as the mean of the absolute ratios of the differences between successive BPs and the minutes between them. Details concerning the TR estimation have been described elsewhere [14]. This parameter focuses on the subsequent changes between consecutive BP recordings, how fast or how slow and in which direction BP values change, and is more sensitive to the sequential order of BP readings than the standard deviation index, which merely reflects the upward and downward BP excursions around the mean.

#### 2.2.2. Cold Pressor Test (CPT)

The CPT is a method commonly used to evoke a sympathoexcitatory stress response [15, 16]. Testing was conducted by trained experimenters in a quiet room. The cap for wireless EEG recording was attached, and the following EEG recording phases were carried out: (i) 3 min resting baseline period; (ii) immersion of the left hand to just above the wrist in a 2°C water bath with eyes closed for 1 min (unit of stress); (iii) continued immersion in the 2°C water bath until the participant, as previously directed, spontaneously withdrew the hand due to intolerability of the cold (pain tolerance assessment); (iv) 3 min resting/recovery phase. Time of immersion, time of 1 min exposure to the cold (“unit” of stress), and time of withdrawal of the hand (tolerance) were recorded. The CPT was well tolerated by all subjects, with no adverse effects noted. EEG monitoring during the CPT: EEG recording was continuous through the 3 min resting phase preceding the CPT, the 1st CPT minute (stress unit), the pain tolerance assessment phase, and the concluding 3 min post-CPT phase.

EEG data collection and analysis: for acquiring the EEG data, the EMOTIV Epoc EEG system was used (EMOTIV, 20141). This device has a wireless amplifier, and 14 wet saline electrodes, corresponding at the positions AF3, F7, F3, FC5, T7, P7, O1, O2, P8, T8, FC6, F4, F8, and AF4 according to the international 10–20 system (see Figure 7).

The device has also an embedded 16-bit ADC which was used to digitize the data with 128 Hz sampling frequency per channel. The data were sent via Bluetooth to a computer with the EMOTIV Control Panel software installed, allowing the visual monitoring of the impedance of the electrode contact to the scalp. The EMOTIV Epoc EEG device is part of a number of low-cost EEG systems, which have been recently applied for research aims. However, recent research assessing their reliability provides converging evidence indicating their capacity to measure consistently EEG signals [17–20].

Two electrodes located just above the subject's ears (P3, P4) were used as reference. Electrode resistance was kept constantly below 5 kΩ. The EEG signals were band-passed filtered with Butterworth 0.5–8 Hz, 8–12 Hz, 12–28 Hz, and 28–45.5 Hz filters.

In order to analyse the data from the experimental setup, a wavelet-based analysis was performed using EEGLAB 13.5.4b [21], an open-source toolbox for MATLAB (Mathworks, Inc., Natick, MA, USA). The data processing in our work is based on the wavelet transform, *W*_*x*_(*t*, *f*), that permits the accurate decomposition of EEG waveforms into a set of component waveforms allowing the isolation of all scales of waveform structure [22]. According to this method, the complex Morlet wavelet is chosen as a mother wavelet, Ψ(*t*), to be convolved with the original signal, *x*(*t*). This convolution leads to a new signal, *W*_*x*_^Ψ^(*b*, *a*), with *b* denoting the translation parameter and *a* the wavelet's scaling parameter. This signal consisted of coefficients which denote the correlation between the EEG signal and the wavelet function. In order to approximate the continuous wavelet transform, the convolution with the signal has to be done *N* times for each scale, where *N* is the number of points in the signal time series. Although the choice of *N* could be arbitrary, given the fact that the exact number of time segment data points of all CPT phases was known beforehand, the appropriate choice of repeated convolutions was made resulting in comparable wavelet coefficients for all time segments. Nevertheless, the wavelet transforms at each scale *a* have to be directly comparable to each other and to the transforms of other time series, so the wavelet function was normalized to have unit energy at all times. The wavelet power spectrum was then computed as |*W*_*x*_(*t*, *f*)^2^| or as |*W*_*x*_(*b*, *a*)^2^| in terms of translation and shifting parameters.

Furthermore, certain coefficients will be generated corresponding to the noise affected zones and some other coefficients will be generated in the areas corresponding to the actual EEG. Although these coefficients are associated with frequency components, they are modified in the time domain, where each coefficient corresponds to a time range. An appropriate choice of wavelet coefficients would result in removing the noisy part of the EEG signal to some extent, while retaining the useful part of the signal [23]. Noise filtering is therefore implemented simply by zeroing out any coefficients associated primarily with noise.

For each electrode the total measurements were divided into four time segments based on the previous described experimental procedure. The wavelet coefficients were split into the following eight standardized bands: Delta (1–4 Hz), Theta1 (4–6 Hz), Theta2 (6–8 Hz), Alpha1 (8–10 Hz), Alpha2 (10–12 Hz), Beta1 (12.5–16 Hz), Beta2 (16.5–20 Hz), Beta3 (20.5–28 Hz), and Gamma (28.5–45 Hz). The wavelet cycles of the transform were dynamically increased so that the time width of the wavelet corresponding to the highest frequency of the Gamma band is to be half the time width of that related to the lowest frequency of the Delta band, thus, allowing a higher frequency resolution (resulting from 3 cycles at 1 Hz to above 67 cycles at 45 Hz). The wavelet coefficients were averaged over time and then scales contained within each frequency band were summed together to yield the absolute activity within each band [12, 24]. For each time segment and frequency band, the Power Spectral Density was calculated by integrating the corresponding wavelet scalogram over time.

## 3. Statistical Analysis 

Statistical analysis was performed with the STATISTICA 12.0 software for Windows. A first analysis involved a between-group, Repeated Measures design. Power spectrum density of EEG recordings from 14 electrodes was expressed as Delta, Theta1, Theta2, Alpha1, Alpha2, Beta1, Beta2, Beta3, and Gamma values. Each frequency was analysed as a dependent variable in separate, 1-way Repeated Measures ANOVAs. In each ANOVA, the independent variable was Group Membership ((1) normotensive controls versus (2) hypertensives); the Repeated Measures factor was phase, which included four levels corresponding to the stages of the Cold Pressor Test ((1) pretest resting phase, (2) stress unit phase, (3) tolerance phase, and (4) posttest resting phase). Special attention was given to interactions as those would provide the strongest evidence as to differential response profiles of hypertensives versus controls. The overall relationship among the clinical and the EEG variables was further investigated by Canonical Analysis; in order to ascertain the relative significance of the variables they showed the highest individual association with the two states of the subjects (i.e., healthy controls and hypertensives). On the left side of the equation we chose those EEG variables that clearly and statistically significant showed an interaction effect via the standard Repeated Measures ANOVA paradigm. Each subgroup of these variables was analysed separately so four Canonical Analyses were carried out, one for each group, that is, Gamma, Alpha1, Alpha2, Delta, and Theta1. On the right side of the equation we chose Maximum Diastolic Blood Pressure of the day (Max DBP day), Minimum Heart Rate of the day (Min HR day), Mean Blood Pressure 24 hours (Mean MBP24), and Minimum Diastolic Blood Pressure of the day (Min DBP day) by performing Discriminant Function Analysis that included all clinical variables and controls/hypertensives as the dependent variable.

We also performed the standard Student's *t*-test to check for differences between the groups regarding the behavioural performance in the pain tolerance of the subjects.

## 4. Results

### 4.1. Behavioural Variables

There was no significant difference between normotensives and hypertensives although a tendency was revealed towards greater pain tolerance in hypertensives measured as self-determined duration (min) of exposure to the cold bath (mean tolerance, controls = 4.62 ± 1.32 min; hypertensives 6.02 ± 2.79 min; *p* = 0.059).

### 4.2. Arterial Blood Pressure Variables

Overall the ABPM variables differed significantly between the two compared groups; however a Discriminant Function Analysis revealed the four most differentiators variables; see Table 2.

### 4.3. EEG Data

A significant main effect of phase was noted in several electrodes (Delta: O2 and F8; Theta1: F3; Alpha1: O2 and P8; Alpha2: F3 and AF4; Beta2: F3 and F4; and, finally, in Gamma electrodes AF3 and F7). Overall, signals tended to rise during the tolerance phase and drop during the posttest resting phase.

However, the central finding was the interactions observed between Group Membership and phase. Analyses revealed interactions in Delta, Theta1, Alpha1, Alpha2, Beta2, and Gamma values. In examining this relationship we encountered four distinct response patterns which support our hypothesis that hypertensives have a differential electrophysiological response profile to environmental stimuli as those are simulated by the phases of the Cold Pressor Test.

#### 4.3.1. Delta Brain Activity (DBA)

**Figure 1:**
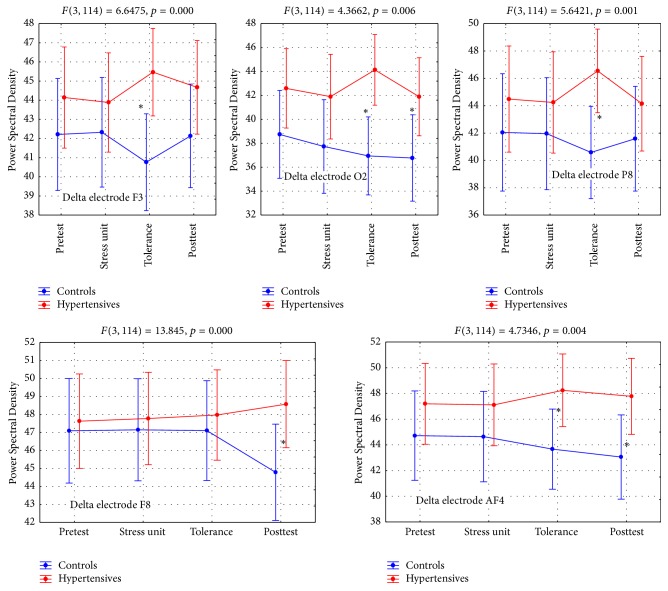
Delta brain activity (DBA). *∗* refers to statistically significant differences.

DBA at F3 and P8 leads had higher values for hypertensives than controls overall and particularly in the tolerance and posttest phases. A similar finding was noted for electrode O2 but with a greater difference between the two groups, with controls showing a continuous value decline from pretest to posttest. In the control group, lead F4 showed a sharp value drop at the posttest phase, whereas hypertensives demonstrated a slight increase at the same phase. In the control group the AF4 lead revealed a steady drop from the beginning of the procedure to the end, similarly to the O2 lead; in contrast, the hypertensives group sustained the same level of activity in all four experimental phases (Table 3, Figures 1 and 7). All five interactions were statistically significant (Table 3).

**Figure 2:**
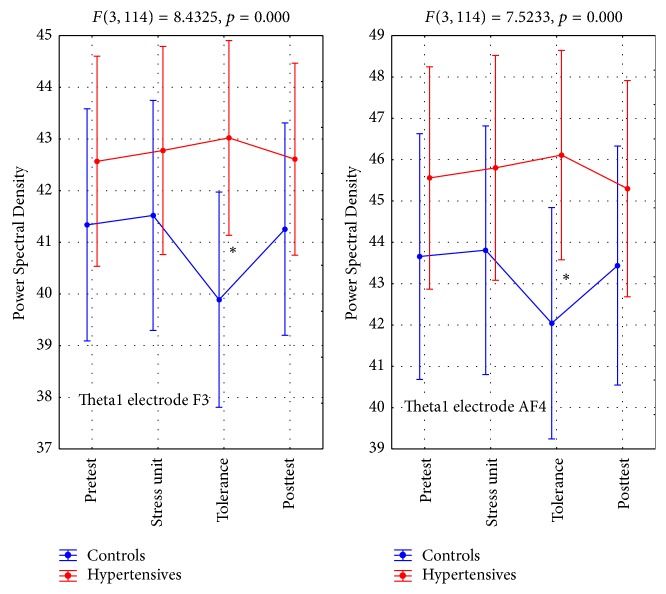
Theta1 brain activity (T1BA). *∗* refers to statistically significant differences.

TBA 1 at F3 and AF4 leads produced a statistically significant interaction (Table 6), with hypertensives showing overall higher values peaking in the tolerance phase. In the same phase controls demonstrated a considerable value drop during the tolerance phase (Table 4, Figures 2 and 7).

**Figure 3:**
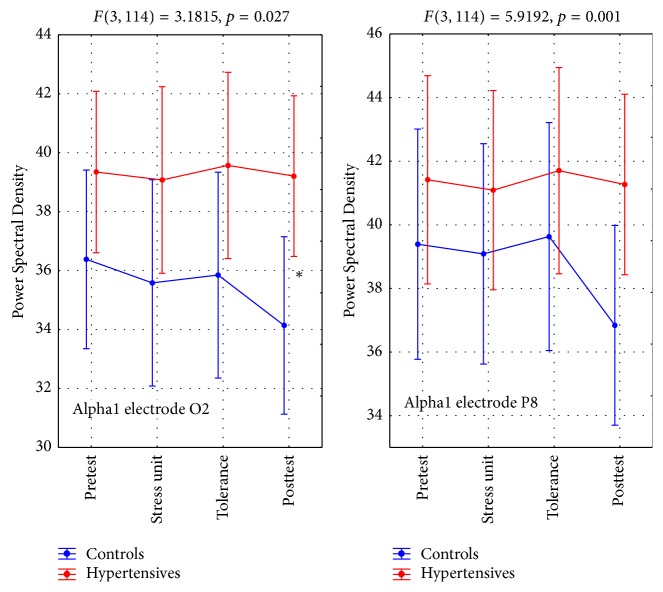
Alpha1 brain activity (A1BA). *∗* refers to statistically significant differences.

Overall, ABA 1 values at O2 and P8 leads were higher in hypertensives than controls throughout the experiment, whereas controls showed a significant drop in the posttest resting phase (Table 5, Figures 3 and 7). This was a statistically significant interaction (Table 5).

In the case of Alpha1 electrode P8 the statistically significant interaction was due to the within the control group drop in the Power Spectral Density value at the posttest phase compared to the pretest (*p* = 0.001) rather than between the groups as noted in most measurements.

#### 4.3.2. Alpha2 Brain Activity (A2BA)

**Figure 4:**
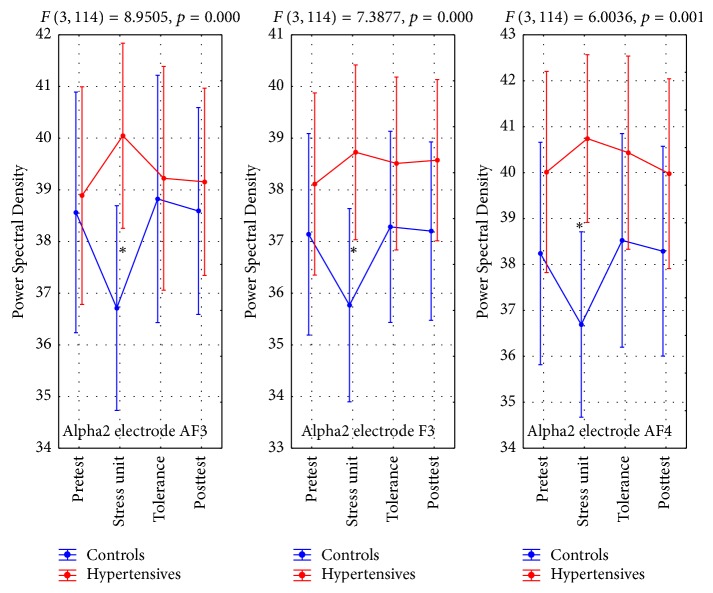
Alpha2 brain activity (A2BA). *∗* refers to statistically significant differences.

A2BA values showed significant interactions at leads AF3, F3, and AF4. The results followed a different pattern from that noted with A1BA. Both groups had similar values during the pretest, with the control group subsequently showing a marked drop during the stress unit phase. In contrast, in that phase hypertensives actually showed a small rise, which levelled off during the tolerance and the posttest phases (Table 6, Figures 4 and 7). These differences were statistically significant (Table 6).

**Figure 5:**
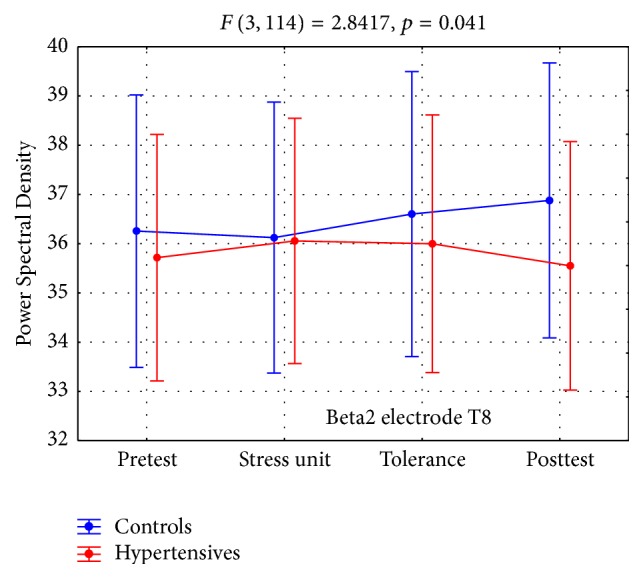
Beta2 brain activity (B2BA).

*B2BA* showed a statistically significant interaction for lead Τ8 (Table 7). Hypertensives had lower values at the pretest resting phase and also at the tolerance and posttest resting phase, compared to healthy controls (Table 7, Figures 5 and 7).

As in the case of Alpha1 electrode P8 the statistically significant interaction in Beta2 electrode T8 was due to the within the control group rise in the Power Spectral Density value at the posttest phase compared to the stress unit phase (*p* = 0.02) rather than between the groups as noted in most measurements.

#### 4.3.3. Gamma Brain Activity (GBA)

GBA values for leads T8, AF3, AF4, and FC6 revealed statistically significant Group X Phase interactions (Table 8). Multiple comparisons indicated AF3 values were lower in hypertensives at the pretest phase and again lower at the posttest phase whereas they were similar to controls during the stress unit and the tolerance phase.

T8 values showed the greatest difference between the two groups during the tolerance phase with hypertensives having higher values than controls.

**Figure 6:**
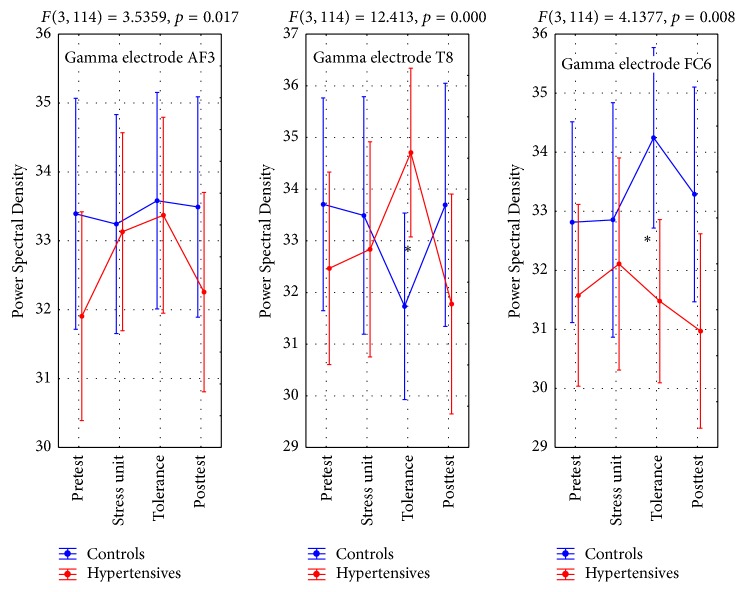
Gamma brain activity (GBA). *∗* refers to statistically significant differences.

**Figure 7:**
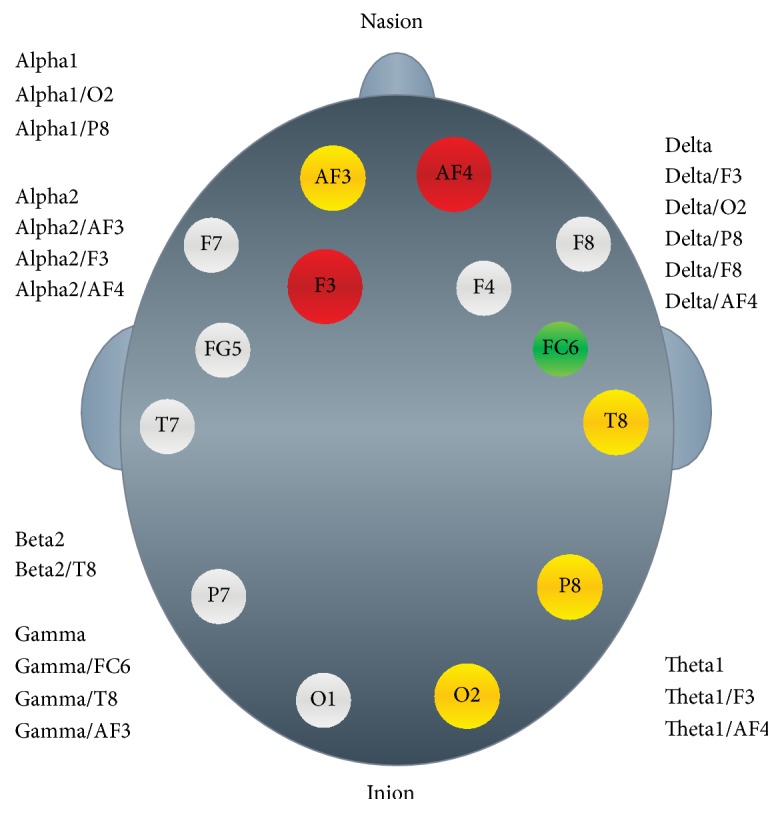
EEG oscillations based on the EMOTIV Epoc EEG apparatus; coloured sites indicate significant group differences.

The electrode FC6 recordings showed a general higher value range for controls especially during the tolerance and the posttest phases (Table 8, Figures 6 and 7).

Once more, the statistically significant interaction for Gamma electrode AF3 was due to statistically significant differences in the Power Spectral Density values what were noted in the hypertensives group who had a sharp rise from the pretest to the stress unit phase (*p* = 0.001) and the tolerance phase (*p* = 0.000) whereas no such changes were observed in the controls group whose values remained similar throughout the entire experiment.

On the left side of the equation we chose those EEG variables that clearly and statistically significantly showed an interaction effect via the standard Repeated Measures ANOVA paradigm. Each subgroup of these variables was analysed separately so four Canonical Analyses were carried out, one for each group, that is, Gamma, Alpha1, Alpha2, Delta, and Theta1. On the right side of the equation we chose Max DBP day, Min HR day, Min MBP24, and Min DBP day by performing Discriminant Function Analysis that included all clinical variables and controls/hypertensives as the dependent variable. Discriminant Function Analysis is useful in deciding which set of variables is best in discriminating between groups of subjects and thus suitable for our purpose in isolating those clinical variables that most strongly predicted membership in our groups.

The obtained results revealed that Delta values where the most strongly associated with the clinical state of the subjects (Canonical *R*: 0.82870; Chi^2^(56) = 67.373 *p* = 0.14224). This was followed by Alpha2 (Canonical *R*: 0.78902; Chi^2^(48) = 64.833 *p* = 0.05315), Gamma (Canonical *R*: 0.75445; Chi^2^(48) = 53.749; *p* = 0.26380), Alpha1 (Canonical *R*: 0.64904; Chi^2^(32) = 32.951 *p* = 0.42043), and Beta1 (Canonical *R*: 0.60100; Chi^2^(16) = 22.403; *p* = 0.13073).

**Figure 8:**
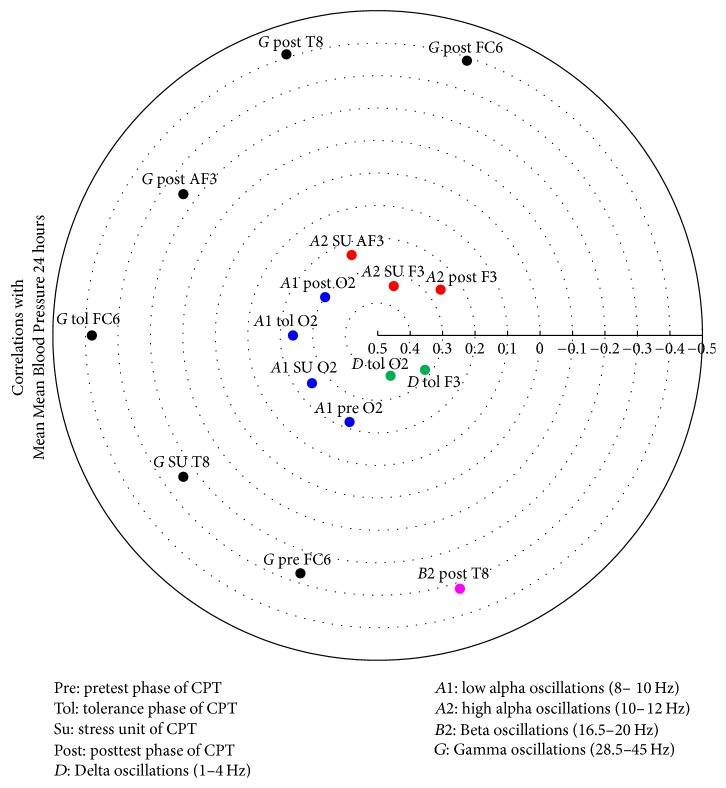
Heliograph of the correlations between Mean Mean Blood Pressure 24 hours and the statistically significant different CPT-induced EEG oscillations depicted by concentric circles in the (−1)-(0)-(+1) continuum.

**Figure 9:**
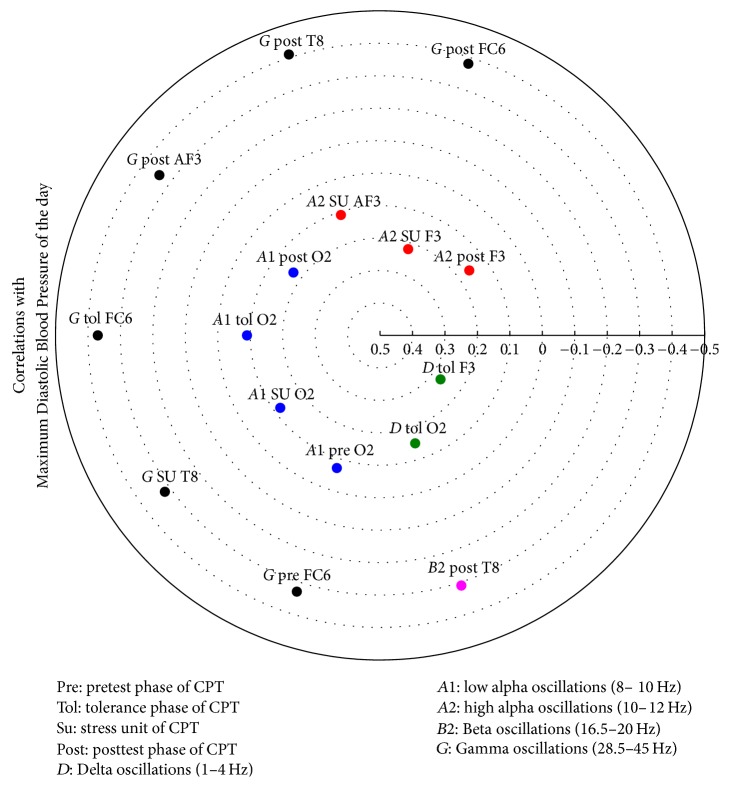
Heliograph of the correlations between Maximum Diastolic Blood Pressure of the day and the statistically significant different CPT-induced EEG oscillations depicted by concentric circles in the (−1)-(0)-(+1) continuum.

**Figure 10:**
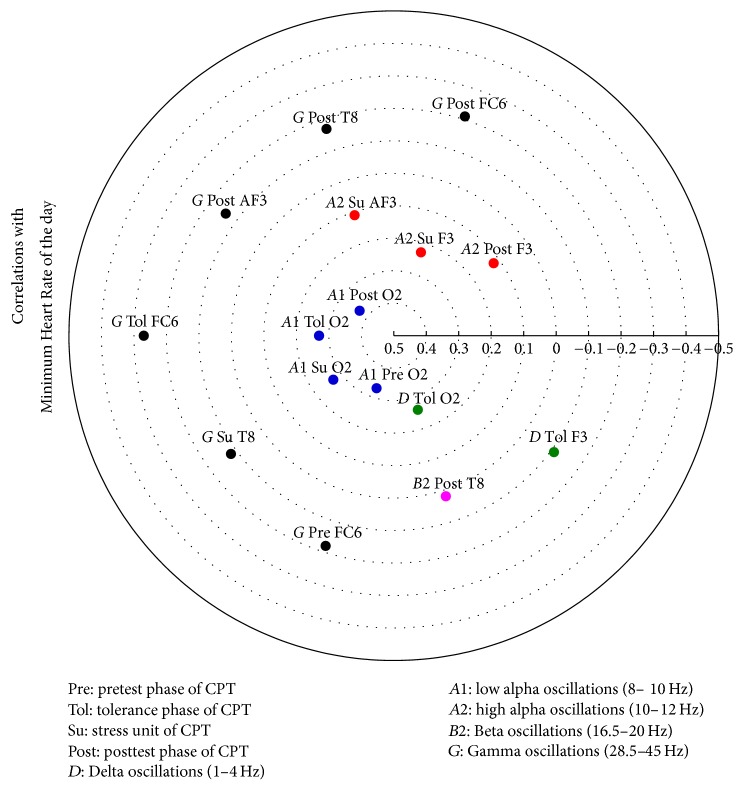
Heliograph of the correlations between Minimum Heart Rate of the day and the statistically significant different CPT-induced EEG oscillations depicted by concentric circles in the (−1)-(0)-(+1) continuum.

**Figure 11:**
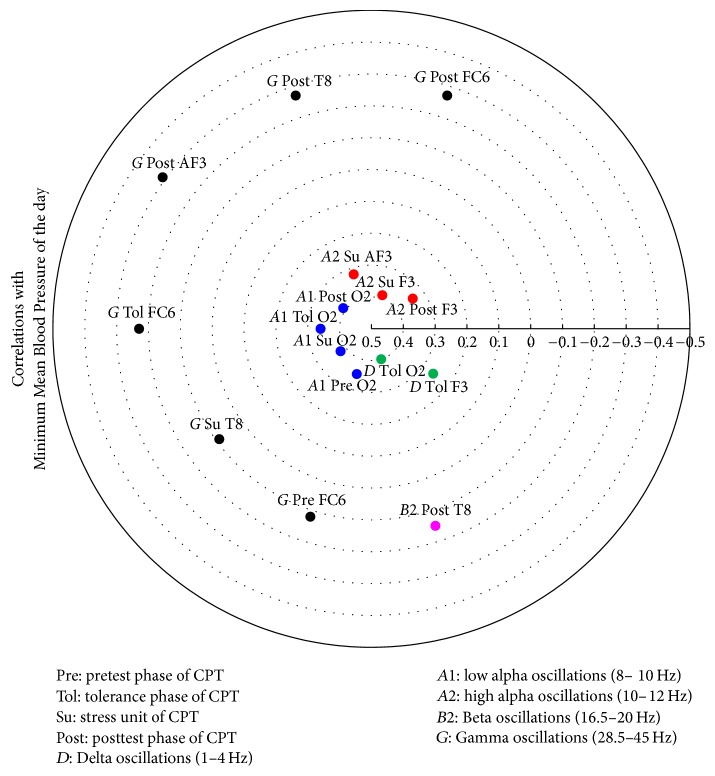
Heliograph of the correlations between Minimum Mean Blood Pressure of the day and the statistically significant different CPT-induced EEG oscillations depicted by concentric circles in the (−1)-(0)-(+1) continuum.

The relevant correlation matrices showing the electrodes involved with correlations higher than 0.3 as derived by the Canonical Analyses are given in Table 9 and in Figures 8, 9, 10, and 11.

## 5. Discussion 

The study explored putative relationships between arterial blood pressure variables (24 hr ABPM) and electrophysiological responsiveness (EEG activity) elicited by exposure to sympathoexcitatory stress/pain induced by the CPT in untreated hypertensives and normotensive controls.

Although the two groups differed significantly in all arterial blood pressure variables, a Discriminant Function Analysis revealed that the most robust group differentiators were four: Maximum Diastolic Blood Pressure of the day (Max DBP day), Minimum Heart Rate of the day (Min HR day), Mean Blood Pressure 24 hours (Mean MBP 24), and Minimum Diastolic Blood Pressure of the day (Min DBP day).

An initial series of ANOVA analyses determined significant group differences in CPT-induced EEG oscillations, which were then covaried with the four most robust cardiovascular differentiators by means of Canonical Analyses. This revealed positive correlations between cardiovascular variables and Delta oscillations (1–4 Hz) during the tolerance phase; in high-alpha oscillations (10–12 Hz) during the stress unit and posttest phase; and in low-alpha oscillations (8–10 Hz) during all four CPT phases.

In contrast, negative correlations were noted between cardiovascular variables and Beta2 oscillations (16.5–20 Hz) during the posttest phase and Gamma oscillations (28.5–45 Hz) during all four CPT phases.

These associations were localised at several sites across the cerebral hemispheres, predominantly in the right one, and in left frontal lobe.

On the behavioural level, pain tolerance measured in terms of self-determined exposure to the CPT ice bath beyond the obligatory 1 min stress unit phase revealed a tendency towards greater tolerance in hypertensives, although this did not reach statistical significance (*p* = 0.059). This is in line with previous findings indicating hypoalgesia in hypertensives. Hypertension-associated hypoalgesia has important clinical implications, the first of which relates to the phenomenon of silent myocardial ischemia and infarcts which are significantly more common in hypertensives than in normotensives [7, 25].

Given the multiplicity of electrophysiological observations based on the initial ANOVAs analyses, for the purposes of this discussion we have focused on the instances where the Canonical Correlation Analysis revealed strong relationships between the cardiovascular and electrophysiological group differentiators of the study.

### 5.1. Delta Brain Activity (1–4 Hz)

The correlations identified between cardiovascular group differentiators and Delta brain activity (DBA) appear compatible with previous human and animal studies suggesting that increased cerebral activity in the spectrum of DBA is associated with increased arterial pressure, probably mediated through suppressed baroreflex control of heart rate [26, 27]. DBA enhancement during perceptual tasks has been associated with functional cortical “deafferentation,” that is, inhibition of sensory bottom-up interferences with internal concentration. It has been proposed that this is a sign of neuronal rearrangement phenomena in the acute and chronic phases of recovery [28]. The sustained increase in DBA activity noted in our hypertensive group is compatible with previous findings suggesting “super efforts” in untreated essential hypertension, possibly due to hypoactivation of the reinforcement system combined with compromised functioning of the brain serotonin system [29, 30].

DBA correlations were noted in the left frontal and the right occipital areas. This is consistent with evidence that DBA is involved in cortical communication over long distances [31]. Yener and colleagues [32] suggested that there were two different networks activated in DBA in response to different stimulus modalities, according to the stimulation characteristics and sensory or cognitive demands.

### 5.2. Alpha Brain Activity, High (10–12 Hz: A2BA) and Low (8–10 Hz: A1BA)

Our findings revealed positive correlations between cardiovascular variables and high (10–12 Hz) alpha oscillations during the CPT stress unit and posttest phases and in low (8–10 Hz) alpha oscillations during the 4 CPT phases overall. The high-alpha subband (A2BA) is considered an index of task-specific sensorimotor activity regulation [33], mainly related to attentional processes [34]: optimal task performance requires inhibition of task-irrelevant areas, which is reflected as high-alpha oscillatory activity facilitating better resource allocation to task-relevant areas [35, 36]. The lower the amplitude of alpha oscillations the better the information transfer through sensorimotor thalamocortical and corticocortical pathways [37]. The CPT is a commonly used method for eliciting primarily sympathetic activation during periods of stress. Hence our finding regarding high-alpha power may underline the positive link between stress and a top-down inhibitory mechanism which, in our study, appears deficient in hypertensives. In line with this view there are findings of increased heart rate and blood pressure in high hostile individuals when exposed to stressful conditions such as the CPT [38].

The low-alpha subband (A1BA) is considered an index of general tonic alertness [33]. The different response of our two groups to pain-related processing may reflect changes of cortical excitability related to the special alerting function of preceding pain [38].

It is a reasonable assumption that the pretest brain oscillations noted in our study may be explained in terms of anticipation of pain. Such anticipation can cause mood changes and behavioural adaptations which may influence subsequent pain perception [39, 40]. It has been shown [34] that the magnitude of a nociceptively induced alpha oscillation related desynchronization (a-ERD) was significantly more dependent on prestimulus than on poststimulus alpha power. A more recent study [41] showed that prestimulus EEG oscillations in the alpha frequency band modulated the subjective perception of painful stimuli: prestimulus alpha oscillatory activities in fact assisted in predicting subjective pain perception. This notion is in line with reports indicating that decreased low-alpha brain oscillations are correlated with higher numerical pain scores collected during both resting-state and noxious conditions by the application of tonic noxious stimuli [42].

### 5.3. Beta2 Brain Activity (16.5–20 Hz, B2BA)

A negative correlation was noted between cardiovascular variables and B2BA during the posttest phase. Given that Beta brain activity plays a role in motor processing, a possible interpretation of this correlation is that pain-related B2BA modulations reflect the preparation and execution of a defensive response [43]. This view is consistent with recent reports showing negative correlations between Beta oscillations and sympathetic activity subserving the interaction between brain neural populations involved in somatomotor control and brain neural populations regulating ANS signals to heart [44].

### 5.4. Gamma Brain Activity (GBA)

A negative correlation between GBA and cardiovascular group differentiators emerged from our study. GBA is considered to play a crucial role in cortical integration and perception [45]. Brief painful stimuli induce GBA in somatosensory cortices [10, 46], probably reflecting local sensory processing in the somatosensory cortex [47].

In contrast, longer-lasting painful stimulation (perception of tonic pain) does not appear to be encoded by GBA in the somatosensory cortex, but rather in the medial prefrontal cortex, close to premotor and cingulate cortices [48]. This GBA encoding of tonic pain might indicate that the subjective perception of longer-lasting pain is more dependent on contextual/emotional processes than on the sensory ones which determine perception of brief painful stimuli. The GBA modulation in our study may be considered in this light.

Previous studies have shown that GBA is enhanced during attentional selection of sensory information [49]. Additionally GBA modulations over the somatosensory cortex are enhanced by attention and vary with pain processing [10, 38, 49]. Thus the enhancement of GBA in our hypertensive group, over the contralateral somatosensory cortex may be related to subjective pain intensity and reflect the internal representations of behaviourally relevant stimuli that should receive enhanced/preferred processing [10, 38]. GBA observed in the pretest (anticipatory) phase of our procedure may be explained in terms of anticipation of pain, causing mood changes and behavioural adaptations which may then influence perception of subsequent pain [50].

In the service of adaptive environmental engagement low coping capacity has been associated with a more pronounced decrease in GBA [51]. Hence the negative correlations obtained between GBA and cardiovascular variables across CPT phases would seem to confirm this perspective. It is compatible with converging evidence which consistently indicates significant involvement of GBA in hemodynamic fluctuations being mediated by the bidirectional connections between neuronal substrates underlying GBA and autonomic responses [52, 53].

As a whole, the correlations we noted between brain oscillations and cardiovascular parameters may be better understood in the light of reports suggesting that cognitive alterations depend upon the degree of hypertension. It is established that the systolic and diastolic blood pressures have effects on distinct cognitive domains [54, 55]. Anson and Paran [56] and Gupta et al. [57] endorsed the view that systolic and diastolic hypertension may also affect cognitive measures in a different way.

Furthermore, the relative scalp locations of differences in magnitude of cerebral activation between the two hemispheres could determine the overall changes in blood pressure and heart rate. This idea is supportive to the view, which states that the two cerebral hemispheres act in concert to promote changes in cardiovascular functioning; however, the right hemisphere predominantly modulates sympathetic efferents, while the left hemisphere predominantly modulates parasympathetic efferents, of the autonomic nervous system [58].

In conclusion, our results add to a growing body of evidence that the brain is implicated in the initiation of high blood pressure while it is itself altered by early disease processes. Thus the brain and vasculature may be independently and concurrently targeted by the factors inducing essential hypertension [59].

Previous studies have thoroughly evaluated the impact of TR of BP variation on target organ damage. A cross-sectional study in 514 normotensive and uncomplicated hypertensive patients demonstrated that the 24-hour rate of systolic BP variation was greater in hypertensive than in normotensive subjects and was the only office or ambulatory BP monitoring parameter that was linearly and independently associated with carotid intima-media thickening [14]. Moreover, another study of our group illustrated that the TR of BP variation, derived from computerized analysis of ambulatory BP monitoring data, is superior to central hemodynamics as an associate of carotid intima-media thickness in hypertensive and normotensive individuals [60]. Additionally, a marker of increased cardiovascular risk, left ventricular mass was linearly and independently related to the daytime rate of SBP variation in hypertensive patients [61]. The former correlation persisted after adjustment for vascular risk factors, body habitus, BP and HR levels, BPV, and nocturnal BP dipping. Furthermore, Manios et al. demonstrated that increased TR of BP variation was independently associated with impaired renal function and coronary atherosclerosis in hypertensive and normotensive subjects, respectively [62, 63]. Moreover, recent studies in acute stroke patients showed that higher TR of BP variation values were associated with brain edema formation and poor outcome [64, 65]. These findings indicate that steeper BP variations may produce a greater stress on the arterial wall and may have an additive role to vascular risk factors and BP parameters in the detection of target organ damage development.

Our results also demonstrate the advantage of simultaneous EEG recording under well-defined pain inducing conditions. Our approach of factoring contributions from multiple, interconnected brain processes is relevant to all studies which attempt to link evoked brain responses with behaviour and demonstrates that exploiting these interactions leads to a more complete understanding of brain response to stimulation and the psychophysiological emergence of the experience of pain.

### 5.5. Limitations

A number of limitations must be considered when interpreting the findings of the current study. First, the study relied on a relatively small sample. Second, we employed CPT as a measure of pain. Although this approach is consistent with the literature, we cannot necessarily generalize the current results to other types of painful stimuli, such as thermal stimuli. Third, an additional limitation relates to the fact that EEG and cardiovascular measurements were noncontemporaneous. This potentially limits the findings in that the two measures were not precisely coupled. Fourth, data were collected in a single testing session. Therefore, we cannot comment on the stability of the relation between hypertension and EEG activity over time.

Nevertheless, the study adds to the understanding of the role of brain oscillations evoked by stress and/or pain stimulation in the underlying mechanisms of hypertension. Our data suggest that brain oscillations in response to stress and/or pain challenge may give greater insight into underlying systems and the mechanisms of hypertension.

## 6. Conclusions

EEG recorded in the course of the CPT provides a measure of cortical activity, on the basis of which untreated hypertensives may be differentiated from healthy controls. Furthermore, the method utilized in this study helps isolate pain-related features in the EEG during CPT in association with cardiovascular variables. This lends credibility to the hypothesis that top-down and bottom-up control mechanisms are implicated in the development of hypertension. Future applications of the methodology may help identify specific EEG features related to the neuronal processing of pain perception in hypertension.

## Figures and Tables

**Table 1:** Outline of experimental measurements.

	Task	Measurements	Duration
Day 1	Habituation to laboratory environment and CPT conditions		1 hour approximately
	Onset of 24 hr ABPM procedure (Section 2.2.1)		24 hours

Day 2	Cold pressor test rest baseline	EEG recording period 1	3 min
	Hand immersion in 2°C water bath	EEG recording period 2 (unit of stress)	1 min
	Continuation of immersion (2°C ) until spontaneous withdrawal	EEG recording period 3 (pain tolerance)	*x* min
	Cold pressor test recovery (Section 2.2.2)	EEG recording period 4	3 min

**Table 2:** 

	Hypertensives		Controls		*p*
	*N* = 22		*N* = 18		
	Mean	SD	Mean	SD	
Mean MBP24	106.19	7.28	91.09	5.95	0.000
Max DBP day	118.59	11.15	98.22	25.97	0.002
Min HR day	62.18	8.87	52.27	16.33	0.019
Min MBP day	85.50	9.92	68.00	19.00	0.001

**Table 3:** 

	*N*	Pretest		Stress unit		Tolerance		Posttest		Interaction
		Mean	SD	Mean	SD	Mean	SD	Mean	SD	
Delta F3										
Controls	18	42.21	4.18	42.32	3.88	40.76	3.49	42.13	3.89	*F*_3_ = 6.65, *p* = 0.000
Hypertensives	22	44.14	7.32	43.88	7.27	45.46	6.39	44.67	6.76	
Delta O2										
Controls	18	38.74	6.83	37.73	7.29	36.94	6.55	36.77	7.34	*F*_3_ = 4.37, *p* = 0.006
Hypertensives	22	42.59	8.31	41.90	8.86	44.13	7.04	41.89	7.73	
Delta P8										
Controls	18	42.05	8.11	41.95	8.12	40.58	6.98	41.58	7.46	*F*_3_ = 5.64, *p* = 0.001
Hypertensives	22	44.48	9.63	44.24	8.93	46.54	7.16	44.14	8.44	
Delta F8										
Controls	18	47.09	3.87	47.15	3.76	47.10	3.64	44.78	3.18	*F*_3_ = 13.85, *p* = 0.000
Hypertensives	22	47.62	7.40	47.77	7.24	47.97	7.09	48.57	6.98	
Delta AF4										
Controls	18	44.71	5.63	44.64	6.03	43.66	5.63	43.05	4.71	*F*_3_ = 7.34, *p* = 0.004
Hypertensives	22	47.19	8.40	47.11	8.30	48.24	7.19	47.78	8.19	

**Table 4:** 

	*N*	Pretest		Stress unit		Tolerance		Posttest		Interaction
		Mean	SD	Mean	SD	Mean	SD	Mean	SD	
Theta1 F3										
Controls	18	41.33	3.40	41.52	3.49	39.88	2.90	41.25	3.34	*F*_3_ = 8.43, *p* = 0.000
Hypertensives	22	42.56	5.54	42.77	5.42	43.02	5.25	42.60	4.95	
Theta1 AF4										
Controls	18	43.65	5.10	43.80	5.79	42.04	4.79	43.43	5.43	*F*_3_ = 7.52, *p* = 0.000
Hypertensives	22	45.55	7.01	45.80	6.68	46.11	6.60	45.29	6.51	

**Table 5:** 

	*N*	Pretest		Stress unit		Tolerance		Posttest		Interaction
		Mean	SD	Mean	SD	Mean	SD	Mean	SD	
Alpha1 O2										
Controls	18	36.38	6.28	35.58	7.26	35.84	7.46	34.13	6.46	*F*_3_ = 3.18, *p* = 0.027
Hypertensives	22	39.34	6.39	39.07	7.39	39.56	7.19	39.20	6.18	
Alpha1 P8										
Controls	18	39.39	7.02	39.08	7.28	39.63	7.11	36.84	5.46	*F*_3_ = 5.92, *p* = 0.00
Hypertensives	22	41.41	8.01	41.09	7.24	41.70	7.81	41.27	7.35	

**Table 6:** 

	*N*	Pretest		Stress unit		Tolerance		Posttest		Interaction
		Mean	SD	Mean	SD	Mean	SD	Mean	SD	
Alpha2 AF3										
Controls	18	38.56	3.93	36.71	4.14	38.82	4.07	38.58	3.70	*F*_3_ = 8.95, *p* = 0.000
Hypertensives	22	38.88	5.52	40.04	4.15	39.22	5.66	39.15	4.55	
Alpha2 F3										
Controls	18	37.13	3.59	35.76	3.48	37.28	3.39	37.20	3.17	*F*_3_ = 7.39, *p* = 0.006
Hypertensives	22	38.11	4.44	38.72	4.23	38.51	4.22	38.57	3.93	
Alpha2 AF4										
Controls	18	38.23	4.16	36.69	3.73	38.52	4.43	38.28	4.77	*F*_3_ = 6.00, *p* = 0.001
Hypertensives	22	40.01	5.69	40.73	4.58	40.43	5.20	39.97	4.79	

**Table 7:** 

	*N*	Pretest		Stress unit		Tolerance		Posttest		Interaction
		Mean	SD	Mean	SD	Mean	SD	Mean	SD	
Beta2 T8										
Controls	18	36.26	4.70	36.12	4.77	36.60	5.10	36.88	5.36	*F*_3_ = 2.84, *p* = 0.041
Hypertensives	22	35.72	6.56	36.06	6.47	36.00	6.75	35.55	6.22	

**Table 8:** 

	*N*	Pretest		Stress unit		Tolerance		Posttest		Interaction
		Mean	SD	Mean	SD	Mean	SD	Mean	SD	
Gamma AF3										
Controls	18	33.39	2.41	33.24	2.40	33.58	2.239	33.49	3.20	*F*_3_ = 3.54, *p* = 0.017
Hypertensives	22	31.90	4.19	33.13	3.92	33.37	3.943	32.25	3.46	
Gamma T8										
Controls	18	33.70	3.44	33.49	4.24	31.73	4.16	33.69	4.96	*F*_3_ = 12.41, *p* = 0.000
Hypertensives	22	32.46	4.90	32.83	5.24	34.70	3.44	31.77	4.89	
Gamma FC6										
Controls	18	32.81	3.32	32.85	3.83	34.24	2.02	33.28	4.21	*F*_3_ = 4.14, *p* = 0.008
Hypertensives	22	31.57	3.74	32.10	4.40	31.47	3.90	30.97	3.44	

**Table 9:** 

Correlations left set with right set	Mean MBP24^1^	Max DBP day^2^	Min HR day^3^	Min MBP day^4^
Delta tolerance F3	***0.32***	0.27	−0.11	0.26
Delta tolerance O2	***0.37***	0.15	0.26	***0.40***
Alpha1 pretest resting O2	0.22	0.07	***0.33***	***0.35***
Alpha1 stress unit O2	0.25	0.12	0.27	***0.38***
Alpha1 tolerance O2	0.24	0.09	0.27	***0.34***
Alpha1 posttest resting O2	***0.30***	0.17	***0.37***	***0.39***
Alpha2 stress unit AF3	0.24	0.11	0.11	***0.32***
Alpha2 stress unit F3	***0.34***	0.22	0.23	***0.39***
Alpha2 posttest resting F3	0.26	0.17	0.12	***0.34***
Beta2 posttest resting T8	−***0.32***	−***0.31***	−0.02	−0.15
Gamma pretest resting FC6	−0.27	−***0.33***	−0.18	−0.12
Gamma stress unit T8	−0.24	−***0.32***	−0.12	−0.09
Gamma tolerance FC6	−***0.38***	−***0.37***	−0.27	−0.23
Gamma posttest resting AF3	−0.24	−***0.34***	−0.14	−***0.31***
Gamma posttest resting T8	−***0.41***	−***0.41***	−0.17	−0.27
Gamma posttest resting FC6	−***0.39***	−***0.38***	−0.21	−0.27

^1^ Figure 8, ^2^Figure 9, ^3^Figure 10, and ^4^Figure 11.
